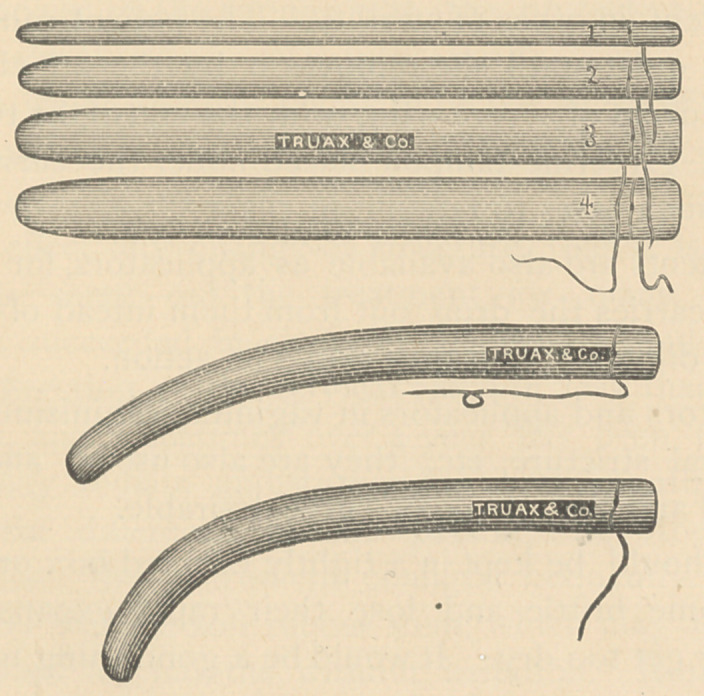# Chicago Gynæcological Society; Meeting of February 18th

**Published:** 1887-04

**Authors:** 

**Affiliations:** 2330 Indiana Avenue


### SOCIETY REPORTS.

TRANSACTIONS OF THE CHICAGO GYNÆCOLOGICAL SOCIETY.

*Regular meeting, Friday, February 18, 1887.*

*I.*—BYFORD. *Slippery Elm Tents.*

*II.*—JAGGARD. *A Placenta, with Marginal Insertion of an Absolutely Short Umbilical Cord, Measuring Nine Inches in Length.*

*III.*—JAGGARD. *The Antiseptic Obstetrical Pads of Dr. H. J. Garrigues and Dr. Wm. L. Richardson.*

*IV.*—JACKSON. *Dermoid Cysts of the Ovary.*

*V.*—FENGER. *Dermoid Cysts of the Ovary.*

*VI.*—JACKSON. *Vaginal Pressure In The Treatment of Chronic Pelvic Disease.*

PHILIP ADOLPHUS, M. D., in the Chair.

I.—DR. HENRY T. BYFORD made the following remarks on

THE SLIPPERY ELM TENT:

For quite a number of years, I have been using slippery elm tents in the treatment of uterine disease. Within the last few weeks, I have received from Messrs. Charles Truax & Co., of this city, some improved tents made out of the compressed bark. By compression, its dilating power is increased so as to render it useful as a substitute for tupelo, sea-tangle and compressed sponge in many cases.

The characteristic of slippery elm bark is that upon being moistened a slippery substances exudes, which acts both as a lubricant to the tent, and as a protection to the mucous membrane against that injury or abrasion which sometimes follows the use of dilators of this class. Another advantage is the rapidity of its action. The tents expand sufficiently within an hour or two for the introduction of a larger size, or two or three of the same size. This may be repeated until the desired dilatation is obtained, and, since the expanded tents are both soft and slimy, with a minimum amount of injury to the parts.

A small elm tent may be introduced at the office and left for twelve or twenty-four hours and thus serve, providing it be used once or twice a week, as a mild substitute for the intra-uterine stem in cases of sterility. The patient can go home and keep quiet until she removes it by the attached string, upon the advent of severe pain, or at the end of the twenty-four hours. A glycerine tampon placed under the cervix is necessary in such cases to retain it in place.

These tents are also available as applicators, for the exuding slime carries the drug out from them intead of into them, gradually dilutes it, and thus limits its action.

As dilators and applicators in vaginitis, vaginismus, urethritis, urethral stricture, etc., they are also useful, and may be ordered of any size, shape or curve desirable.

They should be kept in a tightly covered box or bottle, as they become brittle and lose their rapid expansive power when they get too dry. It would be a good thing to dip them in gelatine or cocoa butter when they are to be preserved for a long time.

DR. A. REEVES JACKSON: I have used slippery elm tents a great many years, and I have no doubt that the form in which they are now presented by Dr. Byford will render them more suitable for dilating purposes than heretofore. In this form, however, I have made use of them recently, and have been disappointed as regards their dilating power. Slippery elm forms a slimy mass at the expense of its substance, and while this mucilaginous mass and the mucous discharge which appears with it causes the tent to seem large, the latter does not exert much dilating power, and I was surprised to find that after moistening one of these compressed tents for several hours in water, it was very little larger than before. I have had a great deal of satisfaction in using flat pieces whittled out of the slippery elm bark, in the following manner: I use a small sponge tent, and in order to get the expansive power of that material surround it with a cordon of these slips, so as to protect the mucous membrane from injury. I think that in the combination of sponge tent and slippery elm we have the best means for tent dilatation. In this way we get the dilating power of the sponge with the protective effect of the slippery elm.

In cases in which stenosis seems to be the cause of sterility, I use non-expansive stem pessaries. My experience in the use of the intra-uterine stem has been very large, and I find, in order to effect any change of structure, that it is usually necessary to carry out the treatment for many months. I have never succeeded in changing materially and permanently the direction of the cervical canal in less than six or eight weeks, and sometimes over a year has been required. The stem may remain undisturbed for many months. This makes a great difference to the patient, who otherwise is obliged to have the instrument changed frequently. I use chiefly a very flexible soft rubber stem, which may be introduced and worn for months without irritation. It may be of different degrees of flexibility, a very small one at first, and of very slight straightening power, and by and by another, larger and stiffer, and so on until at the end of some weeks or months the desired change is produced. I think the use of a substance that is not absorbent and does not dilate superior in these cases.

DR. H. T. BYFORD: I have had many cases of sterility of several years' standing, dating from marriage, cured by three or four or more small tents used once or twice a week. Their power is milder than that of compressed sponge, and less radical than that of the intra-uterine stem pessary. These negative qualities are the accompaniments of nearly all safe remedies. If Dr. Jackson had watched the compressed elm tent, which seemed to expand so little in several hours, he would have found that it expanded two or three times its own diameter in less than two hours, and then became smaller again by having its softer portion dissolved out. In the uterus its substance is not washed away as rapidly as in a basin.

II.—DR. W. W. JAGGARD EXHIBITED A PLACENTA WITH MARGINAL INSERTION OF AN ABSOLUTELY SHORT UMBILICAL CORD, MEASURING NINE INCHES IN LENGTH.

The specimen, placed at his disposal by Dr. Charles Caldwell, was of interest in connection with Dr. John Bartlett's paper, read at the January meeting. The absolute shortness of the cord in Dr. Caldwell's case did not constitute a mechanical hindrance to the progress of labor, although there was some slight difficulty in ligaturing the organ, after birth of the child, on account of the proximity of the navel of the child to the vulvar orifice of the mother.

III.—THE ANTISEPTIC OBSTETRICAL PADS OF DR. H. J. GARRIGUES AND DR. WM. L. RICHARDSON.

Dr. H. J. Garrigues describes the application of the pad in the following words: The well-closed vulva is covered with a pad of absorbent cotton, wrung out in the solution (1,2000). Outside of that comes a piece of oiled silk, or preferably thick gutta percha tissue, dipped in the solution. To keep this antiseptic part of the dressing in place I use a large pad of dry absorbent cotton, and a rectangular piece of canton flannel or a square piece of unbleached muslin, half a yard in both directions, and folded diagonally like a cravat. Dr. Wm. L. Richardson substitutes absorbent scrap or waste done up in cheese-cloth for the absorbent cotton. Of course, Dr. Richardson does not insist upon the pad as essential. There is nothing peculiar about the pad except that it seems to me to be a very convenient and safe form of dressing to use. The main thing is the use of antiseptics all through the delivery, and the pad is all that is needed for the convalescence.

The important services, rendered to the profession and community, in the prophylaxis of child-bed fever, by Garrigues and Richardson, demand recognition. In the New York Maternity Hospital the mortality from sepsis—Oct. 1, 1882, Oct. 1, 1883, 429 patients,—was 6.06 per centum. Garrigues has reduced this mortality to—Oct. 1, 1885, Oct. 1, 1886, 463 patients,—.21 per centum.

In the Boston Lying-In Hospital, the mortality from sepsis, Jan. 1, 1882, Dec. 31, 1882, 288 patients—was 5.55 per centum. Richardson has reduced this mortality to—Jan. 1, 1886, Dec. 31, 1886, 373 cases,—.0 per centum.

The American woman insists upon wearing some sort of a napkin to absorb the lochia. If she wear one at all, it must be antiseptic.

IV.—DR. A. REEVES JACKSON EXHIBITED TWO DERMOID CYSTS OF THE OVARY.

Case 1. First seen by me May 27, 1884. Julia C., aged forty years, was married at twenty, had one child two years later, and no other pregnancy. Commenced menstruating at 17, and was regular after the first year. Always had good health. Five months ago, weighed 176 pounds. Three and a half months ago she took a long walk in the evening, while menstruating. After her return she had a slight chill, and severe pelvic pain, the latter chiefly referable to the bladder, micturition frequent and painful. She seemed never to recover her health; vesical symptoms continued, appetite and nutrition failed; she became rapidly emaciated; a swelling appeared in the lower abdomen; menstruation appeared regularly and without pain. She could not walk, but rode out in a carriage. A few days before I saw her she rode out, with enjoyment, but returned a good deal fatigued and soon began to vomit. Her upper extremities moved involuntarily, and there was a complete loss of power in the lower ones. A "metaphysician" was called to see her and gave the assurance that there was no bodily ailment,—the mind only was at fault. This assurance was repeated twice a day by the Christian scientist, who on these occasions sat out of sight of the patient, the head-board of the bedstead separating them. However, the patient and her friends thought she was getting rapidly worse, and I was asked to see her. At the time of my visit she was lying in bed on her back. Her hands and arms were in constant motion; she seemed powerless to keep them still a moment; she could make no co-ordinate movements, indeed had no control whatever over either the upper or lower extremities; the latter were, however, motionless. The functions of the bladder and bowels were voluntary; the tongue dry and covered with a brownish coating; there was great thirst; pulse 110, small and almost imperceptible at the wrist. The temperature was not observed. She was very restless, and had not slept more than ten minutes at one time during the past three days. At short intervals she vomited a greenish, frothish, tenacious fluid. On examination I found the abdominal walls soft, and free from tenderness on pressure. There was a feeling of doubtful fluctuation with dullness at the sides, also in the right iliac region. The higher parts of the abdomen, as the patient lay on the back, were resonant. She died the following day, and in the evening an autopsy was made with the assistance of Drs. Brower, Danforth and E. Ingals.

On opening the abdomen we found besides other evidences of acute inflammation the exudation of perhaps a quart of pus. As this was being removed it was discovered that its source was a partially collapsed cyst, which still held about one pint of pus, and from the opening through which the pus came there protruded a few hairs. This fact settled the diagnosis. The woman had died of a ruptured dermoid cyst. The kidneys showed evidence of chronic disease. The cyst, with the ovaries and Fallopian tubes, was removed. Here I show you the opening, which was somewhat enlarged after the autopsy for the purpose of freeing the inner portion of the cyst. In addition to the hair there were also some rudimentary teeth and some plates of bones which I sent to a pathologist for examination, and received from him a photograph showing the bony constituents of the cyst. Apparently all of the dermoid tissue was centered in this space. The amount of hair was large, as you see. This was matted together in the usual manner, and has been freed from the sebaceous matter which accompanied it by shaking it in ether. This is a miniature switch made by an artist in such matters from a portion of the hair. These dermoid cysts, of which I have now seen three, possess great interest to me, pathologically. I confess to not understanding them, so I asked Prof. Fenger to be here to-night, and he promised to bring some additional specimens and to talk about them.

Case 11. Dora B., 35 years old, wife of a physician esiding at Normal Park. Saw patient on February 10th 1886. Married fourteen years; two children, aged respectively 11 and 7 years; also two abortions, one three years and the other two months since. Nine months ago, while lying on her back, patient noticed a swelling the size of an orange in the right iliac region. It was soft, smooth, movable and insensitive; has slowly encroached on the opposite side; five days ago, after sneezing, experienced a sharp attack of pain in pelvis which lasted twenty-four hours, keeping her in bed. I found on examination an abdominal tumor occupying the hypogastric and right iliac region, extending upward as high as the umbilicus, and into the left iliac fossa. On February 23d she entered my infirmary and the tumor was extirpated on the 25th. It weighed about eight pounds. There was a main cyst, containing a limpid serum, and inside of this a smaller cyst about the size of a mandarin orange, containing bone and hair. This tumor possesses an interesting feature that I have never observed before in either of the cases I have seen. It is this, there are two distinct kinds of hair, one of an auburn color, very curly, portions of which are still attached to the part, and two tufts springing from other portions of the rough, orange-rind-like skin, which are long and straight, quite free from curl, and of an entirely different color. The kinds of hair that I have seen from other specimens, while they have usually differed in color from the patient's hair, have always been uniform throughout. In the first case I saw, several years ago, the hair was 36 inches long. In that case, the part from which the bony substance and hair sprang were in one part of the tumor, occupying a small space. The patient had carried this for many years without symptoms. Finally it commenced to grow and formed a tumor sufficiently large to attract her attention. There were two cysts. The first one was a very large one, containing a semi-colloid matter very much resembling soft soap, and in great abundance. Separate and distinct from this there was a cyst not larger than a goose's egg, soft, and which could be indented readily, and when it was opened there came out a mass of hair together with other dermoid characteristics. So it seemed that the cyst which contained the fluid and made the growth perceptible was secondary, and this fact would account for the clinical fact of these patients carrying a tumor for many years without any impairment of health until an additional cyst is formed that may present any of the characteristics of an ordinary ovarian cyst.

DR. CHRISTIAN FENGER made the following remarks on

DERMOID CYSTS OF THE OVARY, with illustrations from specimens:

In entering upon the question of the dermoid cysts of the ovary, I wish to call attention to the two theories of their origin. According to Heschl, dermoid cysts in general owe their origin to isolated islands of the epiblast, displaced during embryonal development and located somewhere in the territory of the mesoblast. This theory of fœtal inclusion did not explain the origin of the dermoid cysts in the testicle and ovaries. It was not until His had shown that the internal genital organs are developed from a part of the embryo, the so-called "axenstrang," in which all the germinal layers are included, that we were able to understand the presence of dermoid cysts in those genital glands.

The second theory of the origin of dermoid cysts in the ovary is the view of the older authors, recently adopted by Waldeyer. Epithelial cells of the ovary, capable of transformation into the ovum with all its formative possibilities, may enter into an irregular formative activity and produce a dermoid cyst—a process almost analogous to a partheno-genetic development, as Ohlshausen states it. This second theory would only explain the origin of dermoid cysts in the ovary, and would not enable us to understand their presence in all other parts of the body. Consequently, it seems more natural to accept the Heschl-His theory, as this gives a satisfactory explanation of the origin of dermoid cysts in general, and is in conformity with Cohnheim's theory of the origin of all other new formations, from an isolated group of embryonal cells, dormant until the unknown cause of the new formation calls them into formative activity.

A dermoid cyst is always a monocyst, and if, as is seldom the case, we find more than one in the same ovary (Ohlshausen in one case found three), we may expect to have had more than one embryonal matrix, from each of which a cyst has developed, the one independent of the other. It often appears as if a dermoid cyst of the ovary were a multiple one, but closer examination will prove that we have before us a combination of a dermoid cyst and a proliferating cystoma, or more rarely a dermoid cyst with multiple local colloid degeneration of the stroma of the wall. Cystic transformation of the sweat glands—extensive cysts to the size of a fist—was seen in one case by Friedländer.

I shall not go any further into the subject of the dermoid cysts here, but only present to the Society three specimens removed by laparotomy within the past year, and will call attention to the points of interest illustrated by each one in particular.

Case I. This specimen, at the time of the operation the size of a fist, now much smaller from shrinking in the alcohol, was removed from a girl of 20. There was no difficulty about the removal, but I am sorry to say that the patient died from acute sepsis thirty-six hours after the operation. Besides the sebaceous matter and the hairs, which you have already seen in Dr. Jackson's specimens, we find in dermoid cysts very commonly—in from 20 to 50 per cent. of the cases—teeth inserted in the soft dermoid wall or in pieces of bone contained in the latter, or finally, free in the contents of the sac. As a rule there are only a few teeth in a cyst; but Schnabel has seen, in a case of a girl of 13, over 100, and Autenrieth describes a case in a 22-year-old woman, in which 300 teeth were removed and as many more left in the cyst. As Ohlshausen states, it is impossible to understand the presence of such numbers of teeth without the explanation that, the same as in children, multiplication of the enamel germ takes place and a set of milk teeth are followed by a set of permanent ones. That this is more than a mere theory is proved by a specimen in Rokitansky's collection in Vienna, in which there is seen a milk tooth with the root absorbed down to the crown by atrophy from pressure of the overlying permanent tooth. Spencer Wells, in his "Ovarian Tumors," states that he has seen one similar instance. In the specimen before us this fact is illustrated to perfection. From the soft parts on the surface of this little piece of bone, in the wall of the cyst, you see attached a tooth corresponding in shape and size exactly to a temporary incisor of the upper jaw. I have made an incision through the gum, if we may use that expression, and, as you see, the root is absorbed almost down to the crown. When we turn this milk tooth to the side, we see the crown of the overlying tooth. This is larger, and has the exact shape of the corresponding permanent incisor.

Case 2. The next specimen is a very large dermoid cyst from the left ovary of a woman at 50. It filled the whole abdominal cavity up into the cardia and gave the exact symptoms of a proliferating cystoma or multiple cyst, as there were felt, besides the main cavity, harder, lobulated portions, which I supposed to be smaller and more tense cysts. As she gave the history of a cyst which ruptured when she was 14 years old, and caused several months of suffering from peritoneal symptoms and then disappeared, not to return until after the age of 45, I thought that a dermoid cyst was out of the question. At the operation, which was difficult on account of many adhesions and the nature of the contents of the cyst, I found this very large dermoid cyst, containing—(a) Three or four gallons of a brownish fluid, in which floated hundreds of thousands of round, yellowish-white, small bodies, the size of a hemp-seed up to a pea. I pass round a sample of them in these two glasses. These bodies are soft, have the consistency of butter, and are found under the microscope to consist of irregular masses of amorphous fat, with pavement epithelial cells interspersed here and there, single or in groups.

(b) A yellowish-white, butter-like mass the same as the small bodies if matted together, filling up entirely some of the chambers of the cyst, with no fluid mixed with it. This peculiar arrangement of the fat is rare. Rokitansky saw in a cyst 70 bodies the size of a hazel-nut and very many the size of a pea swimming in a brownish fluid. Routh, according to Spencer Wells, saw a similar case, the balls showing under the microscope concentric layers of amorphous fat around a nucleus of cholesterine crystals. Fraenkel, cited by Ohlshausen, found the whole contents of a dermoid cyst to be numerous hard, mostly round or irregular balls, consisting of amorphous fat, fatty degenerated epithelial cells and hairs. The shape of the cyst is peculiar, inasmuch as it gives the appearance of a conglomeration of cysts. But close inspection shows that all of these communicate with each other so as to form one large though very irregular cavity. Thus in reality we have before us a monocyst, characteristic of the dermoids, as I mentioned before. In the wall, however, we find a number of smaller cysts the size of a pea to a hazel-nut—these do not contain the same fatty material as the main cyst, but a colloid mass, and are due to secondary colloid degeneration in the wall of the latter. The inner surface of the large dermoid cyst shows in some places irregular masses of bone imbedded in the wall, and further, as in Dr. Jackson's cases, the following condition: We do not find typical skin, with hairs, sebaceous glands, epidermis, and so on, everywhere on the inside. This is found only on part of it, forming one or several irregular islands. The remainder of the cyst wall is smooth, has the characteristics of an ordinary cystoma, with a single layer of epithelial, cuboid, or cylindrical cells. It may be that the dermoid portion of the wall secretes the fat and the cystoid portion mainly a serous or albuminous fluid. Movements of a cyst containing at the same time a thin serous fluid and sebaceous matter might (Rokitansky) shape this suspended fat into the small round masses just the same as butter when in the process of churning. However, if this was the right explanation, it appears that this peculiar formation is seen only in very exceptional cases. The right ovary was transformed into a dermoid cyst the size of an orange. Notwithstanding the dermoid cysts on both sides, the woman had a number of children, the youngest 16 years old at the time of the operation. The patient never rallied from the shock of the operation, and died 12 hours afterward.

Case III. The third specimen is a dermoid cyst taken from a girl of 23. It was noticed for about one year and a half before the operation, at which time it was one and a half times as large as a child's head. There was no particular difficulty about gettting it out. When I opened the abdomen and came on the cyst it was transparent, so that I did not think it was a dermoid cyst, and I inserted a Kòberles trocar, which of course we should never do in dermoid cysts if we can help it. Immediately the trocar was stopped up bý what I found later was a mass of hairs and sebaceous matter, so that I had some difficulty in keeping the abdominal cavity clean. However, she recovered without any greater trouble than a little abscess in the abdominal wall from one of the sutures.

Before demonstrating the specimen I wish to make a few remarks in regard to malignancy of dermoid cysts. As a rule we regard a dermoid cyst as a benignant new formation, and a malignant character is here a rather rare exception. We make a distinction between malignancy of a dermoid cyst, *per se*, and malignancy from a combination of dermoid cysts with carcinoma or sarcoma. The malignancy of a dermoid cyst as such is very rarely seen. Kolaczek relates a case, operated upon by Martini, in which besides a common dermoid cyst with a perfectly smooth surface, there was found in the walls of the peritoneal cavity small nodules in great number, the size of a millet seed and of a yellowish color. Many of these little tumors had a light-colored hair sticking out from their centers into the peritoneal cavity. Similar were seen in a case operated upon by Billroth reported by Fraenkel.

Malignancy of a dermoid cyst from combination with carcinoma, sarcoma and myoma: These tumors, originating in the tissues of the cyst, are not so very seldom met with, and have been observed more commonly of late years because a more minute microscopical examination is made now than in former years. Ohlshausen mentions as bearing upon this subject a statement of Doran that he had seen in several instances malignant tumors of the abdominal cavity follow extripation of dermoid cysts. On examining the main wall of the specimen before us, we find on the dermoid island with its hairs and a plate of bone in the wall, the following unusual formations:

(a) A large black mole: It is of irregular lobulated shape, two by three inches in diameter, slightly elevated over the surrounding skin and has a velvety, uneven surface, without hairs. Microscopic examination shows the common structure of pigmented moles, which, as you will remember, has a great similarity to that of a sarcoma.

(b) A papilloma the size of a pea: You will see it outside of the mole on the skin over the bony mass. It is surrounded by a thick wrinkled skin beset with hairs. On transverse section it shows a solid center covered with the pointed excrescences resembling exactly a large wart with long papillæ, as we sometimes find them on the skin of the hand. A detailed microscopic examination and description of all the specimens is not as yet finished, but I intend to give it in a future discussion. It is sufficient, however, here to call attention to the important bearing the two benignant new formations found in this cyst have upon the malignancy just spoken of. It is well known that moles often furnish the soil for sarcomas, and that warts or papillomas for years benignant, sometimes all of a sudden commence to grow because they are transformed into a carcinoma or a sarcoma. The rapidity with which a dermoid cyst sometimes will grow involves a great nutritive hyperactivity. I can understand that this, in its turbulent way of forming tissues without an aetiological object, could cause the physiological resistance to disappear and thus open up the gates for malignant tumors.

IV.—A. REEVES JACKSON read the following paper, entitled :

VAGINAL PRESSURE IN THE TREATMENT OF CHRONIC PELVIC DISEASE.

The brief paper which I have to present this evening was suggested by some remarks with which the society was favored at its last meeting, by Dr. Etheridge, entitled a "Preliminary Note on Antiseptic Tamponnement of the Vagina in the Treatment of Pelvic Inflammation."

It would have afforded me pleasure to endorse the treatment which was advocated at that time, had an opportunity been given for so doing, for I have had occasion to make frequent use of it, and to learn its advantages, during the past eight or nine years.

My attention was first called to this subject by reading a paper which was published by Dr. V. H. Taliaferro, of Atlanta, Ga., in 1878, on "The Application of Pressure in Diseases of the Uterus," in which the writer presented many facts and arguments to prove the great therapeutic efficacy of the principle of pressure as applied to the treatment of diseases of the uterus and other pelvic organs, which are characterized by habitual passive congestion and its results, namely, uterine displacements, enlargement, relaxation, cervical erosions, menstrual disorders, etc.

The method consisted in firmly packing the vagina with sheep's-wool made antiseptic with carbolic acid, with the aid of a Sim's speculum, the patient being in the knee-chest posture.

At first Dr. Taliaferro used cotton pledgets, saturated with glycerine; but, observing that the cotton packed quite hard, he very soon substituted wool because of its resiliency, a quality which it was found to retain under pressure and moisture.

In illustration of the results of this method of treatment he detailed a number of instructive cases in which it had been used by him.

In one of these the patient was suffering from supra-vaginal elongation of the uterine cervix, complicated with complete cystocele and vaginal eversion, the involuted parts protruding from the vulva and forming a tumor of considerable size. The uterine canal measured six inches. The parts were restored and the vagina packed with cotton, a process which was repeated every two or three days for a fortnight, at the end of which time the depth of the uterus was reduced to three inches. Other symptons were correspondingly improved. The patient, who had been only able to drag herself along with pain and difficulty, could, after the first packing, move with rapidity and comfort.

She was subsequently cured by a plastic operation on the vagina.

A number of other cases, some of them furnishing results almost equally striking, were detailed by the writer.

Dr. T. strongly emphasized the importance of applying the tampon with the patient in Sim's position, in order that the vaginal canal should be distended and elongated to its utmost capacity. He further advised that the first few pieces composing the tampon should be of cotton, for the reason that a greater amount of glycerine may be incorporated with that substance than with wool. It was claimed that the therapeutic effects of this treatment are as follows:

1. It diminishes blood supply and nutrition.
2. It promotes absorption.
3. It removes hyperplastic tissue by retrograde metamorphosis.
4. It diminishes nervous action.
5. It rectifies malpositions.

I was much impressed by the stated results of the treatment, and determined to give it a trial. It seemed to promise a valuable substitute in some of the objectionable and uncertain methods of local treatment then and now in vogue, such as cauterization, local blood-letting, tents, intra-uterine medication, iodine painting, hot douches, etc.

Since then I have used it in many cases of chronic pelvic disease, and am able to corroborate the favorable statements that have been made concerning its efficacy.

Dr. P. F. Mundè, who gives an abstract of Dr. Taliaferro's paper in his "Minor Surgical Gynæcology," edition of 1885, says: "Of the value of this steady elastic pressure and support in reducing the size of an engorged hyperplastic or (better still) subinvoluted uterus, and restoring the normal circulation to the edematous and congested pelvic cellular tissue, I have no doubt whatever; neither of the potent alterative effect of this pressure on old peritonitic or cellulitic exudations and adhesions."

I had not applied this dressing many times before I observed occasionally on removing the tampon that on various parts of the vaginal wall, and also around the os uteri, erosions appeared, sometimes bleeding slightly on exposure. I attributed this to the fact that the packing had either been too firmly or unequally placed.

In cases of moderate laceration of the cervix uteri, this accident is especially likely to occur if the packing is so applied about the vaginal portion in such a manner as to widely open the os uteri. Hence, in all such cases I endeavor to at first push the uterus upward with a single pledget, and then to pack the entire vaginal fornix about it so as to press the cervical labia together as much as possible.

When any part of the mucous membrane appears soft and succulent, I have found advantage in combining with the glycerine a solution of tannin or alum.

The contact of glycerine is not equally well borne by all vaginas, and in a few cases I have not been able to persist in its use on account of the irritation it caused. In these cases I find an excellent substitute in vaseline, which, although it does not produce the peculiar serous drain which comes with the use of glycerine, is unirritating, and makes possible the employment of the pressure, which is the more important element in the treatment.

When I first began to use this pressure treatment I chose carded wool, in accordance with the suggestion of Dr. Taliaferro. But it was difficult to obtain a well prepared article, and next to impossible to incorporate any considerable quantity of glycerine with it. I was obliged to use cotton for the upper part of the vagina. I next tried successively oakum and jute.

These substances were elastic—especially the former—and also antiseptic : the former containing tar, and the latter carbolic acid. However, since sheep's-wool has been so prepared as to be free from fatty matter, and is comparatively absorbent of water and glycerine, it more completely and perfectly meets the indications than any of the other substances I have named.

As regards the form of the tampon, I have used it both in single and multiple pieces, and unhesitatingly give preference to the latter in many cases. It is very important that the vagina be packed in such a way as to insure an equable pressure against every part. This cannot be so certainly done with a tampon made from a single piece, or a few large ones as with a number of smaller sizes. When moistened the pieces should not exceed a walnut in size. Time may be saved, however, and the object accomplished, by using a single piece of wool for the lower half of the vagina.

Commonly, the only medication I have used with the tampon besides the glycerine or vaseline, has been the occasional addition of tannin or alum. But when, for any reason, I have wished to have the dressing remain longer than two days, I have, after saturating the separate pledgets, rolled them in boracic acid so as to take up two or three drams of the latter.

The cases in which I have found this method of treatment especially beneficial are those which are characterized by soft engorgement—such as the earlier stages of subinvolution, with or without cervical laceration. In these cases I have seen more marked change effected in two weeks than is commonly seen in two months—or more than is seen at all sometimes—under the use of hot water douches, however perfectly and assiduously the latter may be used.

Permit a slight digression: Without wishing to disparage in the least the use of hot-water irrigations in the treatment of chronic pelvic inflammations, I desire to say that for some years I have held the opinion that their efficacy, great as it is, has been overrated. Indeed, they have been so eulogized that perhaps we have expected more from them than was reasonable. One serious drawback to their usefulness arises from the fact that the sittings cannot be continued usually for a sufficiently long time. If it were practicable to keep a stream of hot water playing against an inflamed or engorged tissue for thirty hours rather than thirty minutes, we should doubtless obtain more prompt and more permanent results. But as the hot-water douche is usually employed, its effects in constricting the over-full vessels are of short duration. I have seen a turgid, purplish cervix subjected to a hot stream for forty minutes; at the end of the time it was pale and shrunken; at the end of another hour, the patient continuing meanwhile on her back, I have found the same cervix as turgid and as purple as before.

Now, just on this account, a manifest and very great advantage may be urged in favor of a means of treatment which, equally with the hot-water douche, has power to unload the vessels of their stagnant contents, and which may be continued day after day and week after week, without remission and without reaction. Such a means is, I believe, to be found in this persistent pelvic pressure and tissue drainage.

Were it needful I could cite many cases illustrative of the beneficial effects of this treatment, but will content myself with but two.

Case I. A married woman, 34 years of age, had two children at term, and subsequently a miscarriage at the fifth month. After this latter event menstruation became more profuse and the periods were protracted. At the end of two years her general health was greatly impaired and she was markedly anæmic. Ordinary remedies were used without success. At my suggestion, her physician curetted the interior of the uterus, and then swabbed the cavity with Churchill's solution of iodine. Febrile symptoms followed, and lasted a week. Temporarily there was improvement as regarded the hemorrhage; but in three months she was worse than before, and rarely free from a bloody discharge. It was then determined that I should repeat the curretting. Remembering the inflammatory sequel to the previous operation, I was moved to pack the vagina a few times as a preparatory measure. She was flowing when the first packing was placed. When the latter was removed at the end of forty-eight hours, the only appearance of blood was a slight staining of that portion of the tampon which had been pressed against the os uteri. Another tampon, larger than the first, was placed, carrying the uterus as high as possible in the pelvis. On its removal two days later no blood at all was perceptible. This treatment was continued three weeks, combined with suitable medicinal and hygienic means, with the result of permanently stopping the hemorrhage, and the ultimate restoration of the patient's health.

Dr. Mundé, in speaking of this means of treatment in connection with another class of cases, uses these words: "When the retro-displaced fundus uteri is adherent, these daily emollient and hydrogogue tampons may in time, by their combined pressure and alterative action, bring about the absorption, or at least stretching, of the adhesion, and permit a replacement of the organ." I submit a case in point.

Mrs. J., aged 24 years; had several induced abortions; no child at term. Had been treated for displacement by pessary, with apparent benefit. After a time the symptoms returned, and the physician introduced a larger instrument. It caused pain at once, and in a few hours there was a chill and then rise of temperature. I saw the patient next day and advised the removal of the instrument, which was taken away. It was a very large one. A sharp attack of inflammation ran its course in ten days. No abscess formed. A few weeks later I found the uterus retroverted and the fundus immovably fixed by adhesions in its mal-position. At the request of the attending physician I then took charge of the patient. The treatment consisted wholly in the use of tampons of cotton with glycerine and boracic acid. The pledgets were small at first, and were placed in the posterior vaginal fornix, pressed into position with as much force as the patient could readily bear. The pledgets were increased in size and others were placed in front of the cervix. The vagina was packed below more and more fully and firmly each time with wool, until the canal was distended to its utmost capacity. At first the dressing was renewed daily, then every two days. At the end of two months the uterus was thoroughly replaceable, all tenderness had disappeared, and no evidence remained of the former presence of adhesions.

DISCUSSION.

DR. PHILIP ADOLPHUS: In the treatment of chronic pelvic disease by vaginal pressure, we may avail ourselves of the two methods of massage and columning the vagina. The latter has a much wider range in the treatment of pelvic disease than massage.

These methods have been hitherto applied to the removal of congestions, exudates, and recent slight adhesions of the serous tissues in the pelvis, which were within reach of vaginal pressure.

We had often thought that we had succeeded in removing by them old adhesions and bands, when merely recent effusions surrounding old deposits had become absorbed, just as nature will, without our aid, absorb a large pelvic effusion in a recent pelvic inflammation.

Neither method, however, can cause the removal of old cicatricial bands, firm adhesions and imbedded organs; and both are contra-indicated when inflammation of the serous tissues exists.

But where dilatation and congestion are present, and comparatively recent adventitious and hyperplastic tissues are to be removed, the stimulant and alterative influence of pressure on the pelvic vessels induces absorption by either of these methods.

The treatment by massage will not, in future, be resorted to as often as formerly; for it is inefficient in its methods, dangerous in its tendency, as well as troublesome and indelicate to the physician and patient.

The sole object of massage is to induce sufficient irritation in order to effect absorption. But the tamponnement of the vagina does much more than massage.

It supports and relaxes tense ligaments; elevates the movable or adherent vagina, bladder, uterus and ovaries, provided they are not adherent to the pelvic walls; depletes congested, inflamed and subinvoluted organs; overcomes spasm and irritation, and induces physiological rest in the parts.

Tamponnement per vaginam is therefore indicated in all cases where pelvic tenderness is present which is not due to an acute attack, or where absorption is needed; it is efficient in cases of mal-position and prolapse of the uterus, ovaries, ligaments and vagina, where elevation of the organs and mechanical support are required, preparatory to the use of pessaries, or where these cannot be borne.

Columning the vagina is effected in the knee-elbow position by means of Sim's or Simon's speculum. A large pinch of iodoform, boric acid or salicylic acid is first applied to the cervix, a few tampons saturated with glycerine are laid in the vault of the vagina, and then ordinary cotton wool, absorbent wool, or iodoform gauze, is systematically packed into the vagina, to remain there for three or four days. This packing is to be renewed until the effects are produced which the practitioner desires. The patient is not obliged to remain in bed, and the pelvic, sacral and hypogastric pains, together with urethral irritability, are frequently relieved in a short time by this method, which is altogether a more successful, cleanly and decent mode of procedure than that of massage.

DR. JAMES H. ETHERIDGE: I have nothing additional to say except that the continued use of this method in many selected cases has produced most desirable results. But I would protest most emphatically against being understood as recommending it for every trouble of a pelvic nature. For the class of cases Dr. Jackson has enumerated I think it a vastly superior treatment. I was much impressed with the article of Dr. Taliaferro. He tampons the uterus cavity, with the patient in the genu-pectoral position, using a speculum of his own device, which is flanged at the lower end so as to separate the posterior portion of the vaginal orifice, the cervix being held steadily down by the vulsellum, and with a long-toothed forceps he pushes the cotton into the uterine cavity. The true explanation of the benefit which comes from this treatment is, that by elevating the uterus the pressure from its great weight is relieved. There is a mechanical obstruction to the return of blood from the uterus, and what is done by the tampon is to push up the uterus and permit its decongestion, and along with that comes the improved nutrition of the organ, and the reflex symptons in the way of pain, menstrual disturbances and the like readily disappear. I cannot tell exactly how I was put upon this method of treatment; I don't claim it as anything new. Several years ago Dr. Bozemann of New York tamponned the vagina, calling it "columning the vagina." He spoke of several cases of positive elongation of the posterior wall of the vagina with this continual pressure. His paper was published in full in the transactions of the American Gynæcological Society.

DR. H. T. BYFORD: I agree with Dr. Etheridge, that any pressure that can be made by the vaginal pack could not cause the relief. The benefit of pressure upon enlarged veins in any part of the body, as in the leg or testicle, is only temporary unless some other curative influence be added. Nor do I think that a low position of the uterus causes the venous stasis, for this is not found in all cases, and often is found when the uterus is held high up by contracted and indurated sacro-uterine ligaments. The veins are large, long and tortuous, and are made to admit of considerable change in position of the uterus in almost any direction. The rapid improvement comes from the support to the uterus, and sometimes also to the ovaries, taking away the traction upon inflamed and indurated ligaments, and thus promoting the absorption of exudations that either diminish the caliber of the veins or prevent them from accommodating themselves to the position of the uterus and its anexa. This relief of strain and promotion of absorption in the pelvic tissues is the great remedy for subinvolution in the subacute stage, the same as rest in bed is the remedy in its acute stage, viz.: soon after labor. It is in the subacute stage of pelvic disease that the vaginal pack finds its great sphere of usefulness. Dr. Jackson's second case serves as a good illustration. When the inflammation and induration are in the sacro-uterine ligaments, two or three soft glycerine tampons, made of the best ordinary cotton batting (not the absorbent), placed under and in front of the cervix every second day at the office, and left till the next night, will often relieve the traction and bring about rapid improvement. When it becomes necessary to apply the complete pack, we will get the best effects by so placing the cotton and cotton wool as to relieve the traction upon tender parts, which should be found beforehand by a careful diagnosis.

DR. JACKSON: I think the subject has been very thoroughly discussed and the principle of the treatment clearly illustrated. The important fact is that it is not a difficult method of treatment; that it is efficacious there can be no doubt—the clinical facts justify this assertion. The method pursued by Dr. Bozemann seems to me to be peculiarly objectionable. Strapping a woman on to a machine for the purpose of packing the vagina, seems both irksome and unnecessary. I am very glad there is such unanimity of opinion in regard to the clinical efficacy of this method of treatment.

W. W. JAGGARD, M.D., *Editor.*

2330 Indiana Avenue.

## Figures and Tables

**Figure f1:**